# Spring frost risk for regional apple production under a warmer climate

**DOI:** 10.1371/journal.pone.0200201

**Published:** 2018-07-25

**Authors:** Christian Unterberger, Lukas Brunner, Stefan Nabernegg, Karl W. Steininger, Andrea K. Steiner, Edith Stabentheiner, Stephan Monschein, Heimo Truhetz

**Affiliations:** 1 Wegener Center for Climate and Global Change, University of Graz, Graz, Austria; 2 FWF-DK Climate Change, University of Graz, Graz, Austria; 3 Swiss Federal Institute for Forest, Snow and Landscape Research WSL, Birmensdorf, Switzerland; 4 Institute for Atmospheric and Climate Science, ETH Zurich, Zurich, Switzerland; 5 Department of Economics, University of Graz, Graz, Austria; 6 Institute for Geophysics, Astrophysics, and Meteorology/Institute of Physics, University of Graz, Graz, Austria; 7 Institute of Biology, University of Graz, Graz, Austria; INRA - University of Bordeaux, FRANCE

## Abstract

Spring frosts, as experienced in Europe in April 2016 and 2017, pose a considerable risk to agricultural production, with the potential to cause significant damages to agricultural yields. Meteorological blocking events (stable high-pressure systems) have been shown to be one of the factors that trigger cold spells in spring. While current knowledge does not allow for drawing conclusions as to any change in future frequency and duration of blocking episodes due to climate change, the combination of their stable occurrence with the biological system under a warming trend can lead to economic damage increases. To evaluate future frost risk for apple producers in south-eastern Styria, we combine a phenological sequential model with highly resolved climate projections for Austria. Our model projects a mean advance of blooming of –1.6 ± 0.9 days per decade, shifting the bloom onset towards early April by the end of the 21st century. Our findings indicate that overall frost risk for apple cultures will remain in a warmer climate and potentially even increase due to a stronger connection between blocking and cold spells in early spring that can be identified from observational data. To prospectively deal with frost risk, measures are needed that either stabilize crop yields or ensure farmers’ income by other means. We identify appropriate adaptation measures and relate their costs to the potential frost risk increase. Even if applied successfully, the costs of these measures in combination with future residual damages represent additional climate change related costs.

## Introduction

Temperate climates are characterized by seasons. Woody and perennial herbaceous plants have adapted their life and growing cycles accordingly. Late spring frost that coincides with bud break may severely damage the sensitive emerging leaves and flowers. Hence, the timing of bud break is substantial for trees. Freezing events exert a strong selective pressure and trees have adjusted spring phenology to delay flushing until the likelihood of frost occurrence and severe damage to emerging tissue is small [1,2]. A long growing season, however, is essential to maximize growth [3].

Trees thus have to manage the split between flushing too early with a high threat of being severely damaged by late spring frosts and flushing too late, resulting in a reduced growing period. Autochthonous trees evolved safety margins, a sufficient time period separating budburst from the period of occurrence of spring frost [1,2,4], and therefore seldom experience spring frost injury to developing leaves and flowers [5].

However, orchard trees, such as apple, are often not indigenous and cultivated varieties may not be perfectly adapted to avoid damage by late spring frosts [5]. Apple blossoms are easily damaged by frost and substantial yield losses occur when apple flowering and spring frost events coincide, with a single frost being sufficient to damage the sensitive apple blossom [6].

As a consequence of increasing spring temperatures, apple blooming in Europe has occurred considerably earlier over the last thirty to forty years with an average advance of 2–3 days per decade [5,7,8]. Analyzing whether this advance has caused an increase in spring frost risk in the last decades, a recent study from Switzerland reported such an increase only for apples growing at higher elevations (> 800 m, [5]), while for lower regions in Switzerland and other regions in Germany it remained more or less constant [5,9]. For Northern Italy a slight decrease in frost risk was observed [10].

Looking into a future under further climate change, projections for the evolution of spring frost risk for apple production are controversial. While for areas in Great Britain [11] and Finland [12] an increase in frost risk is projected, no significant changes are projected for areas in Northern Italy, France and Germany [8,10,13]. Comparable predictions are also available for other crops, e.g., vine [14,15].

Different factors determine the future development of spring frost risk, which partly work in opposite directions. Increasing spring temperatures significantly advance leaf unfolding and flowering [8,16,17]. This advance, however, might be counteracted by a delay in the break of bud dormancy due to mild winters, resulting in an insufficient fulfillment of chilling requirements [16,18]. As a consequence, these biological processes imply that on the one hand a further overall warming might actually slow down the advance of flowering, leading to no change or even a slight decrease of spring frost risk [5,10]. On the other hand, following fulfilled chilling requirements, flowering shifts into a period of the year in which cold conditions occur more frequently, resulting in an increase of spring frost risk [5,14].

One weather regime that can trigger cold conditions in winter and early spring is meteorological blocking, i.e., stable high pressure systems that block the regular atmospheric circulation and favor advection of cold air from the north [19–22]. Past observations indicate a decrease in winter blocking, while blocking in spring has increased in recent decades [23]. For the future, model projections suggest a further decline in the winter blocking frequency until 2100, but with considerable uncertainty [24]. For the future evolution of blocking in spring, hitherto, there is a general lack of studies.

Current climate models underestimate blocking by as much as 50% [25], implying that spring frost risk could be substantially higher than climate modelling studies indicate to date. While future changes in phenology, i.e., the development of the advance in leaf unfolding or flowering, have been intensively studied [8,10,13,16], a discussion of the contribution of blocking to spring frost events in this context is missing in the literature to date. Accordingly, in this study we can build upon the former, but put our focus on the latter, to close that gap in our understanding of the development of spring frost risk in a warmer climate.

**Fig 1:**
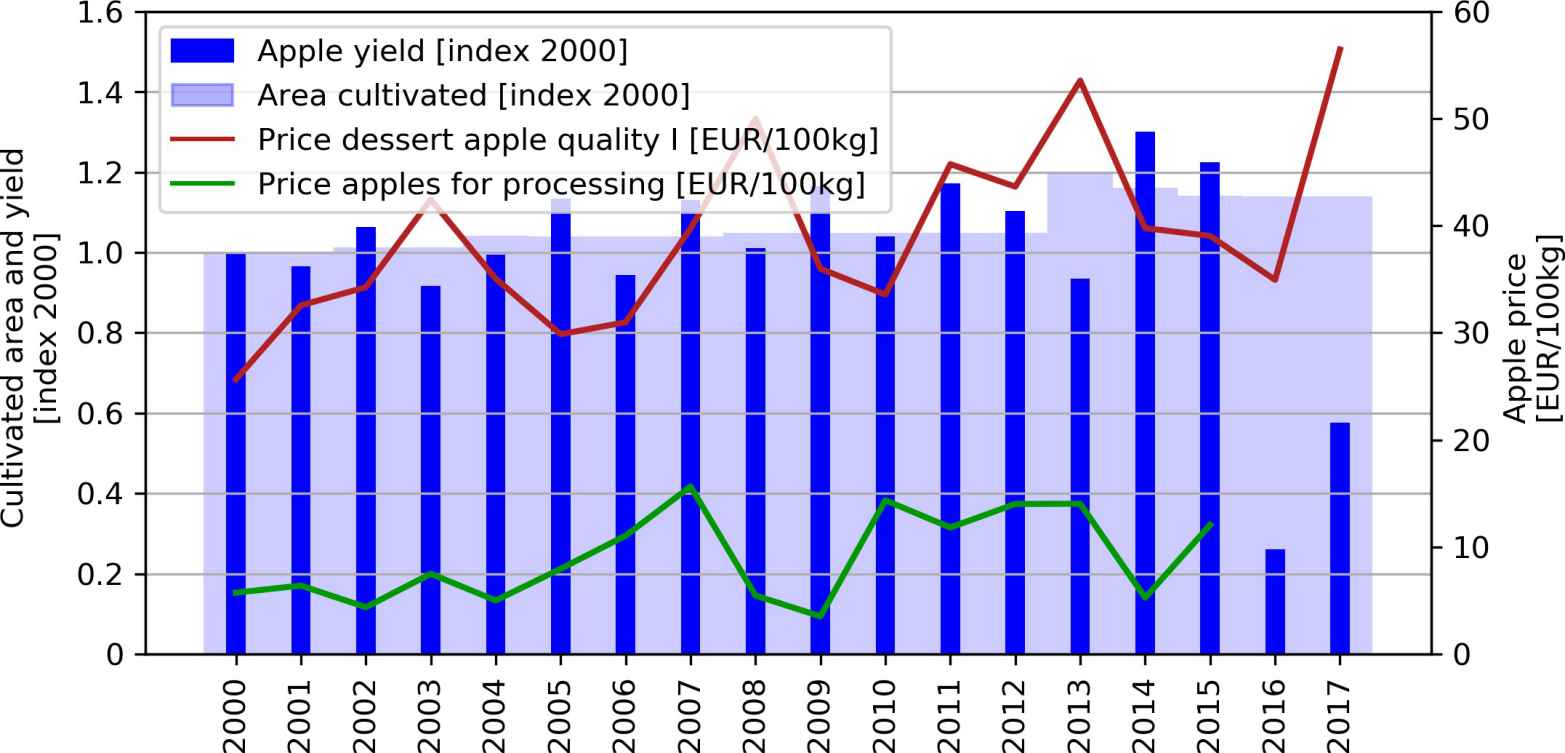
Apple production and prices in Austria from 2000 to 2017 (based on [26]). The prices for apples for processing are only available until 2015.

In April 2016, severe spring frost afflicted Central Europe, including the province of Styria, the main apple-producing region of Austria. Above-average temperatures in early spring led to early blossoming and a succeeding blocking-induced cold spell (April 25 to April 30, 2016) caused direct losses to fruit, wine and vegetable producers of EUR 215 Million (M) in Styria (about 18% of the Styrian, and 3% of the total Austrian agricultural output value in 2016 [26]). Almost 80% of the Styrian apple crop failed [27,28]. In April 2017 unusually warm temperatures again led to an early blossoming of cultivars, which once more fell victim to a cold spell (April 20 to April 24), this time, however, attributable to radiation frost [29]. Direct losses exceeded EUR 50 M for (mainly) Styria and the neighboring regions Burgenland and Carinthia [30]. The crop failure in 2017 amounted to around 40% (Fig 1).

Apples represent around 80% of the annual Austrian fruit harvest (in tons, excluding wine). In 2015, 216,092 tons of apples were harvested, with 177,002 tons originating from Styria [26]. Thereof, the majority of apples are Golden Delicious (27%) or Gala (25%). Other varieties like Idared, Jonagold and Braeburn have shares below 10% [31]. Clearly, events like the ones experienced in April 2016 and 2017 have detrimental effects in a region as specialized to one agricultural product as south-eastern Styria.

Despite the harsh decrease in the regional quantities produced, apple prices did not increase in response to the crop failure in 2016 (see Fig 1). While within Austria, Styria supplies around 80% of the apple production, it is dwarfed at the European level, with countries like Poland, Romania, Italy and France cultivating the vast majority. Due to a strongly connected intra-European market, Styrian apple farmers have no influence on prices. Normally any local undersupply is quickly filled by foreign supply. Regional crop failures faced by Styrian farmers, even if substantial, are therefore not automatically counterbalanced by higher apple prices. The cold spell in 2017 though, not only affected Austria but also the main apple producing countries in Europe and reduced the European apple production by around 20%, compared to the three year average production volume. This widespread crop failures triggered a significant price increase [32] (Fig 1).

To adapt their businesses to frost risk, apple farmers can engage in physical, cultivar and economic adaptation. Physical adaptation protects the crop yield by means of frost protection sprinkling, forced air circulation, artificial heating, or abandoning unfavorable areas. Cultivar adaptation means changes in the cultivated varieties. Economic adaptation ensures the stability of farmers’ income via insurance and/or diversification.

With respect to physical adaptation, frost protection sprinkling is currently considered the most effective measure. Cultivated areas are constantly sprinkled with water as soon as the humid temperature reaches 0°C, protecting cultivars until –6°C [33]. The downside of this method is its high water consumption as around 4,500 m^3^ of water are needed to protect 10 ha of crops against frost for three consecutive nights [34,35]. This excessive water consumption warrants the ample availability of water, a requirement which has to be considered, particularly in regions for which an increase in drought risk [36] and subsiding ground water levels are projected.

Forced air circulation is particularly effective in preventing damage from radiation frost [37]. Wind machines and helicopters are used to mix air vertically and transport it horizontally [38]. The success of forced air circulation depends on the inversion strength (i.e., the temperature difference between cool air near the surface and warmer air layers above) and is limited to frost events with temperatures above –4°C [37]. For artificial heating, anti-frost candles or gas-powered heating machines are installed alongside the vulnerable cultivars.

Aside from these physical measures, the abandonment of unfavorable, exposed cultivated areas coul󐀠reduce the impacts of cold spells on apple farming. This would counteract a recent development, as since the mid-1970s increasingly less favorable areas, situated mainly in basins and therefore more exposed to frost risk [39,40] were cultivated. With respect to cultivar adaptation, a change of the composition of cultivated varieties towards more frost resistant varieties could further limit the impacts of frost events.

Economic adaptation is possible by purchasing insurance against crop failure. In Austria, frost insurance can currently only be purchased in conjunction with hail insurance (Österreichische Hagelversicherung). At present (i.e., in response to the 2016 frost event), premiums are publicly subsidized by 50%. Aside from this, farmers can reduce the proportion of income generated by plain apple production by offering more products with higher value added (e.g., ciders, spirits and dried fruits). Typically, profit margins on refined products are higher and the phenotype of apples is less important. Additionally, it is also possible to provide specialized services (e.g., bottling and filling services and storage), but due to high investment costs and a restrained market in Austria, the prospects of these measures are limited.

Here, we investigate the frost risk faced by Styrian apple producers and aim to estimate the economic risk arising from its uncertain evolution under future climate change. To evaluate the development of frost risk for apple blossom buds, we project apple blooming by using a phenological sequential model [5,41], calibrated (and validated) with observational data [42], and force it with the most recent highly resolved Austrian climate projections. We consider potential frost impacts and explicitly investigate the impact blocking events have on spring frost risk. Our economic evaluation builds upon the damages experienced in spring cold spells in 2016 and 2017, where regions in Central Europe lost more than half of their fruit harvest, assesses available adaptation options and relates their costs to the frost risk faced by Styrian apple producers.

## Data and methods

**Fig 2:**
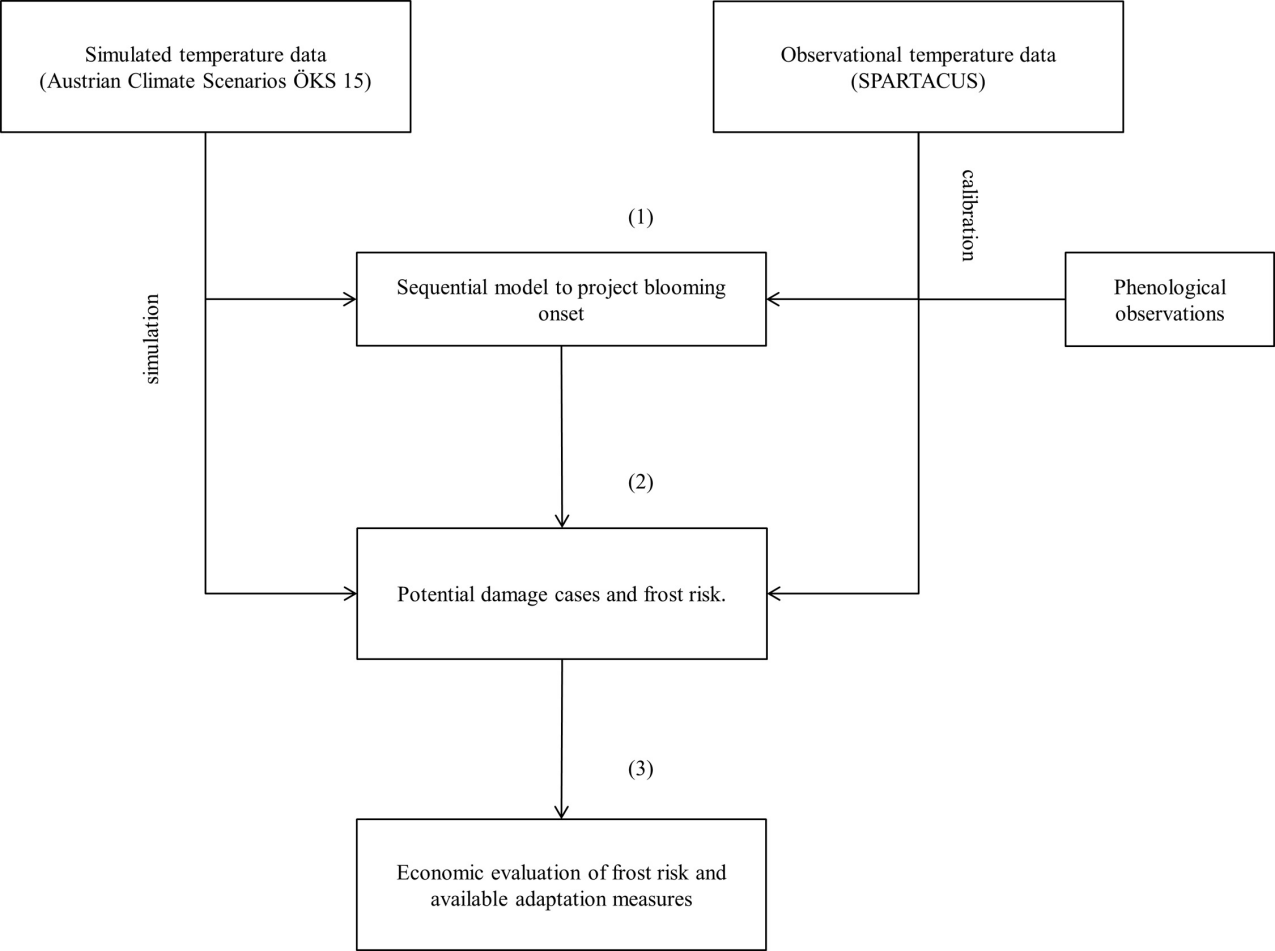
Schematic structure of the analysis. We follow a three step approach that starts with the projection of blooming onset by applying a sequential model, calibrated with observational data and driven by simulated temperature data. Second, potential frost damages are identified. Third, frost risk and available adaptation measures are evaluated in economic terms.

To investigate the evolution of spring frost risk faced by apple producers in south-eastern Styria under the projected climate change, we follow an interdisciplinary approach, which incorporates data and methods from meteorology, biology and economics (see Fig 2). We apply observational and simulated temperature data to a sequential model to project future apple blooming (marked by (1) in Fig 2). Potential frost damages are identified when the projected blooming dates interfere with a temperature threshold of –2.2°C [43] (2). Afterwards, the losses as experienced in 2016 and 2017 are examined in connection with the income distribution of Styrian apple producers and the costs of the adaptation measures are contrasted to the annual risk faced by apple producers (3).

### Sequential model to determine apple blooming

To determine the beginning of apple tree blossom (flowering stage 61 BBCH) we use a sequential model (see Eqs 1–4, and M2 in [41]), calibrate it with observational data, and force it with simulated temperature projections. Starting on August 1 every year, daily mean temperatures *T*_*i*_ between 0.0°C and 10.0°C are converted to chilling rates *R*_*c*_, with a base temperature for chilling *T*_*Bc*_ of 4.2°C [41]:

$$R_{C}\left(T_{i}\right)=0,\;if\;T_{i}\leq 0.0\;or\;T_{i}\geq 10.0$$

$$R_{C}\left(T_{i}\right)=\frac{T_{i}}{T_{Bc}},\;if\;0.0<T_{i}\leq T_{Bc}$$ (1)

$$R_{c}\left(T_{i}\right)=\frac{T_{i}-10.0}{T_{Bc}-10.0},\;if\;T_{Bc}<T_{i}<10.0$$

These chilling rates are summed up until the state of chilling *S*_*c*_(*t*) reaches the chilling requirement C* at day of the year *t*_1_:

$$S_{c}\left(t\right)=\sum_{i=t_{0}}^{t}R_{C}\left(T_{i}\right),\;where\;S_{C}\left(t_{1}\right)=C^{*}$$ (2)

Once the chilling requirements are fulfilled, daily mean temperatures *T*_*i*_ above the base temperature for forcing *T*_*BF*_ of 1.1°C are converted to forcing rates *R*_*f*_:

$$R_{f}\left(T_{i}\right)=0,\;if\;T_{i}\leq T_{BF}$$

$$R_{f}\left(T_{i}\right)=\frac{28.4}{1+exp\left(-0.185\left(T_{i}-T_{BF}-18.4\right)\right)},\;if\;T_{i}>T_{BF}$$ (3)

These forcing rates are summed up until the state of forcing *S*_*f*_(*t*) reaches the forcing requirement F*:

$$S_{f}\left(t\right)=\sum_{i=t_{1}}^{t}R_{f}\left(T_{i}\right),\;where\;S_{f}\left(t_{2}\right):=F^{*}$$ (4)

We calibrate the parameters of the sequential model to historic (1961 to 2006) phenological observations in the Weiz region (south-eastern Styria, Austria) [42] using temperature observations from the SPARTACUS data set [44]. We choose a chilling requirement of C* = 30.5, which corresponds to an average break of dormancy at day of the year 19 [41] and a forcing requirement of F* = 182.5 by minimizing the root-mean-square-error (RMSE) between simulated and observed blossom dates.

SPARTACUS is an observational data set with daily temperature values from 150 homogenized surface stations in Austria and its surroundings that are interpolated onto a highly resolved grid with 1 km x 1 km grid spacing [45]. Weiz is considered representative as it is in close vicinity to many apple producers in south-eastern Styria and therefore shows similar conditions. At the same time the station in Weiz has the longest available phenological time series in this region (1961 to 2006).

We simulate the onset of apple tree blossom in the region from 1952 to 2100 with the calibrated phenological model, based on simulated temperatures from regional climate models. We use 11 high-resolved Austrian climate projections with daily time steps. These national projections (Austrian Climate Scenarios ÖKS15 [46]) are based on the latest generation of regional climate projections from the European branch of the Coordinated Downscaling Experiment [47] of the World Climate Research Programme, EURO-CORDEX [48]. It has been brought onto the Austrian grid (1 km x 1 km grid spacing) by means of a novel statistical bias-correction method, in order to increase the confidence in the regional climate change information provided [49]. However, since the effects of climate change on the dynamics of the atmosphere, including blocking, are uncertain in climate models [50], the associated uncertainty remains in these regional projections. The applied regional climate models as well as their driving global circulation models (GCMs) are summarized in S1 Table in the supporting information.

Additional to the sequential model, we also apply a thermal time model (more specifically M1 in [41]; see the supporting information for a description). Based on an evaluation of the evolution of the state of chilling on December 31, we assume for this model, that the chilling requirements continue to be fulfilled also in the future (S1 Fig in the supporting information) and sum up forcing rates starting from January 1 every year.

### Definition of spring frost risk

Potential damage cases, i.e., interference of frost with blossoming, are identified in a given year if the minimum temperature falls below –2.2°C within 10 days after the date of blossoming [43]. To ensure robustness of our results, we consider the 25 grid points in the 5 km x 5 km region around Weiz and calculate potential damages for each grid point.

To analyze the amplifying effect atmospheric blocking potentially has on spring frost risk, we establish a link between meteorological blocking events and regional spring cold spells, based on observational data from south-eastern Styria. We compute blocking based on data from the European Centre for Medium-Range Weather Forecasts (ECMWF) Reanalysis Interim (ERA-Interim) [51] and use a standard blocking detection algorithm based on 500 hPa geopotential height gradients [52] detailed in Brunner et al. [22]. Blocked days are defined if blocking is found in the Euro-Atlantic blocking region (30.0°W to 45.0°E and 45.0°N to 72.5°N). We furthermore use daily minimum temperature observations from SPARTACUS for detecting days with temperatures below –2.2°C in an area of at least 10 km^2^ in the south-eastern Styrian region (15.4°E to 16.1°E and 46.8°N to 47.0°N).

For periods of 20 days in all springs between 1979 and 2011 we then compare the occurrence of frost days (i.e., temperatures below –2.2°C) during blocked days and during random drawn days, respectively. To adjust for the high autocorrelation of days with blocking we draw clusters of random days (i.e., *N* days with blocking are compared to *N* random days with the same auto-correlation).

### Economic assessment of adapting to spring frost risk

To assess the frost risk faced by Styrian apple producers from an economic perspective, we analyze agricultural production and price data provided by Statistics Austria [26,53]. In particular we look at the distribution of the average income per apple producer between 1991 and 2017 to determine the probability of income reductions as severe as experienced in 2016 and 2017.

Whilst the income losses in 2016 and 2017 both can be considered as rare events, apple farmers are persistently confronted with a varying annual income. This is attributable to a combination of annual variations in apple yield and changes in apple prices (see Fig 1). To get an idea about the annual risk apple farmers face with respect to income losses, an exceedance probability curve is constructed that ascribes a probability to each income loss. The average annual income loss (AAL) faced by farmers is described by the probability-weighted sum of all possible income losses or the integral of the exceedance probability curve [54,55].

We consider the estimated AAL faced by apple producers as a threshold to evaluate the available adaptation measures. Each adaptation measure entails costs. Insurance contracts require the annual payment of premiums to receive coverage, whereas frost protection sprinkling calls for water availability and functioning installed equipment. The ideal adaptation measure prevents as many sources of income losses as possible at an annual cost below the AAL.

To compare the adaptation measures with each other, we look at the total annual cost of each measure for the average cultivated area per apple producer in Styria (i.e., 4 ha). We consider the annual total costs for 0, 1, 3, 5, and 10 frost nights per year and compare it to the AAL. The total annual cost for each of the adaptation measures consists of fixed costs (investment costs for equipment and infrastructure) and variable costs (fuel, working hours, maintenance). The costs for the different adaptation measures considered are presented in S2 Table in the supporting information.

### Data sources

The climate indicator scenario ÖKS15 data are available under a Creative Commons Attribution Share-Alike license [46]. The observational data from the SPARTACUS dataset [44] is provided by the Zentralanstalt für Meteorologie und Geodynamik (ZAMG, Austria).

Historic phenological observations were provided by the Pan European Phenology Project PEP725 [42]. It is an open and universally available database.

Data on Austrian apple production and prices was obtained from Statistics Austria [26,53,56] and is freely available under the links provided in the references. Information on the adaptation measures described and their specific costs are obtained from the references listed in the introduction section and S2 Table in the supporting information, respectively.

## Results

### Blooming and frost risk projections

**Fig 3:**
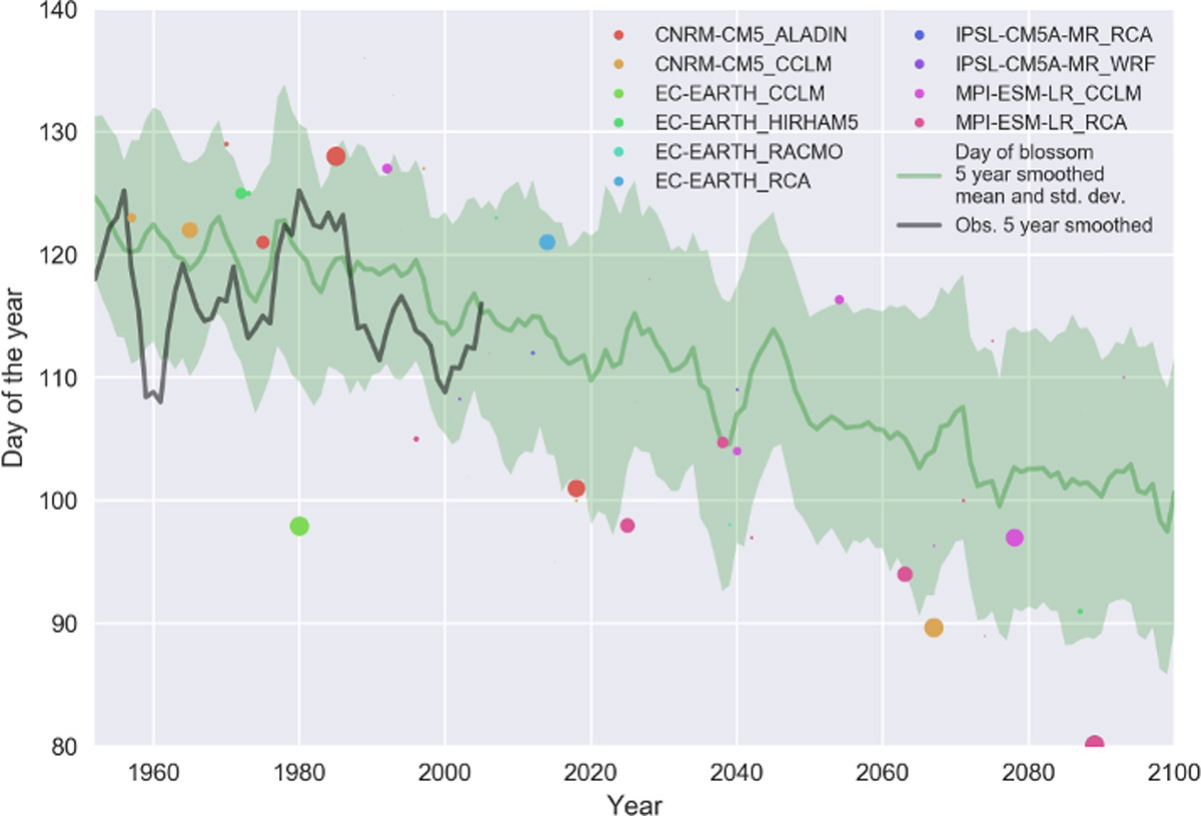
Evolution of the blossoming day of apple in Weiz from 1951 to 2100 from the 5-year smoothed multi-model mean (green line) plus standard deviation (green shading) as well as from phenological observations from 1951 to 2006 (gray line). Potential frost damages (temperatures below –2.2°C occurring within 10 days after blossoming) are indicated for different regional climate models under RCP4.5 (colored dots). The dot size indicates how many grid points around Weiz are affected. Note that climate projections are not initialized with recent climate observations so that the variability of the simulated blossoming in the historical period does not match the observations.

Our phenological model projects a mean change of –1.6 ± 0.9 days per decade for the start of apple blossoming in the Weiz region, considered representative for south-eastern Styria. As shown in Fig 3, apple blossom onset is found to shift to early April in the second half of the 21st century for a moderate global warming scenario, the Representative Concentration Pathway with a radiative forcing increase of 4.5 W/m^2^ relative to pre-industrial values (RCP4.5) [57,58]. This represents a somewhat larger shift than found by Hoffmann and Rath [8] of about –1.3 days per decade. They report a change in the onset of bloom of –12.9 ±3.3 days calculated as the difference in the 30-year mean 2071–2100 compared to 1971–2000, however, with a distinct variability on a regional scale.

Fig 3 also indicates potential frost impacts (colored dots). With respect to their evolution, we find that impacts are highly variable but overall we do not find any robust change in damage occurrence in the moderate (RCP4.5; Fig 3) or extreme (RCP8.5; not shown in Fig 3) warming scenarios, as the earlier blossom period is paralleled by a shift of earlier last frost days. The results from the thermal time model (S2 Fig) reconfirm these results, showing a similar evolution of blooming.

However, the general under-representation of atmospheric blocking in climate models, which has been noted by many studies [25,52,59], may lead to an under-representation of frost events in the simulation runs. This becomes especially relevant if the blossoming onset moves further towards early spring, since here the impact of blocking on cold conditions in Europe is particularly strong [20–22]. Especially for winter, a robust connection between blocking and cold extremes in Europe also in a warming climate has been shown by many studies [20,21,60,61]. The underrepresentation of blocking in the climate projections in Fig 3 may therefore underrate the risk of future frost damage.

In contrast to climate projections, observational studies under current climate change point to an increase in blocking occurrences in spring, resulting in more extreme weather [23,62,63]. Investigating temperature observations between 1979 and 2011, Fig 4 reveals a statistically significant higher occurrence of frost days during blocking in winter and early spring, which is particularly strong in March to April until about day of year 110. This highlights the relevance of the separate statistical analysis of blocking-induced cold spells from observational data.

**Fig 4:**
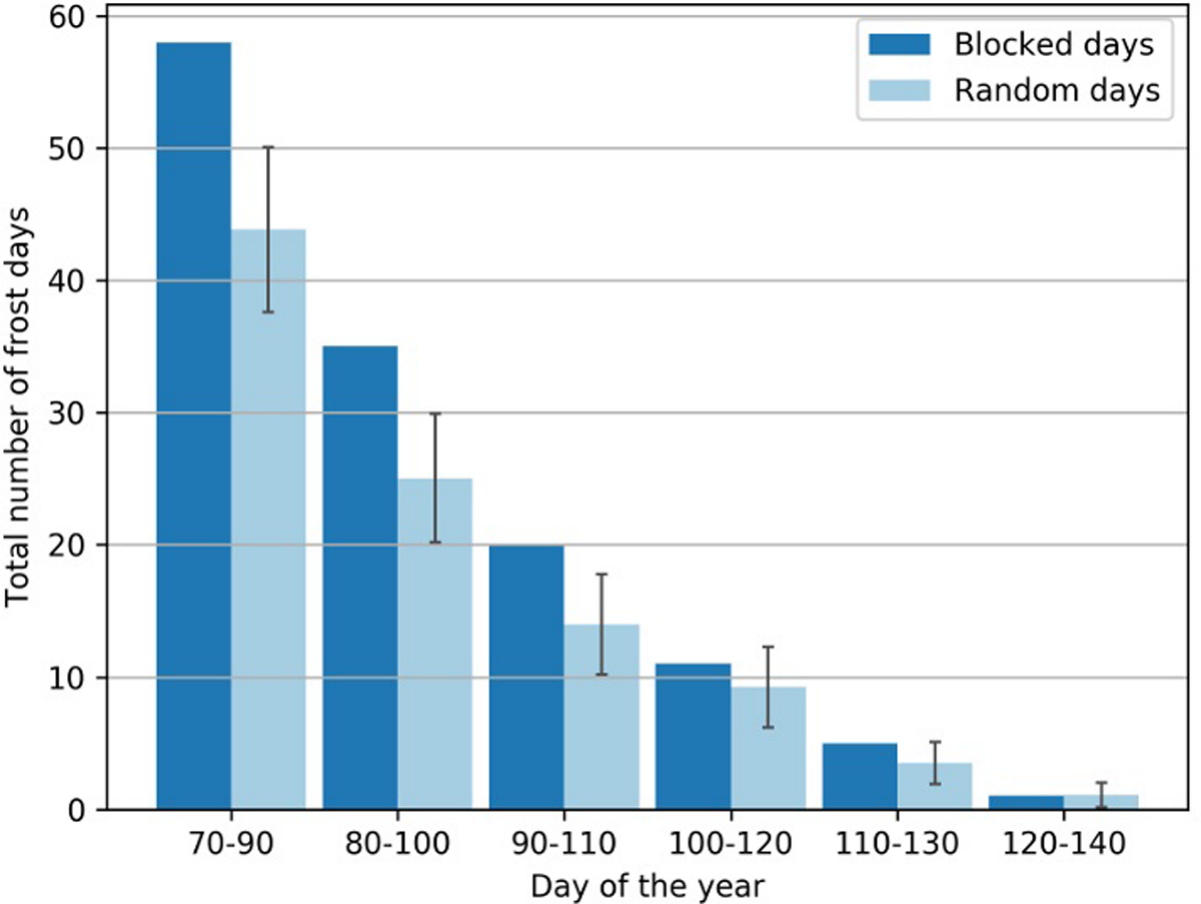
Number of frost days (temperature below –2.2°C) coinciding with blocked days (dark blue) as a function of the day of the year. Number of frost days during randomly drawn days, mean (light blue) and standard deviation (black bars) based on a Monte Carlo test with 1000 repetitions.

In addition to blocking, other weather situations can lead to frost events as well. In April 2017, a cold front brought air from the north to Austria, leading to large amounts of snowfall in northern Styria, followed by radiation frosts in south-eastern Styria. Overall, such other weather situations and their impacts are highly variable. However, over the past decades, a cooling trend has been observed in Eurasia during winter while the Arctic was warming. This can be largely explained by a shift of the stratospheric polar vortex to more frequent weak states which are linked to cold surface extremes in mid-latitude regions [64] and can lead to anomalous cold winters despite Arctic amplification and to changes in mid-latitude weather patterns [65].

### Economic assessment of adapting to spring frost risk

**Fig 5:**
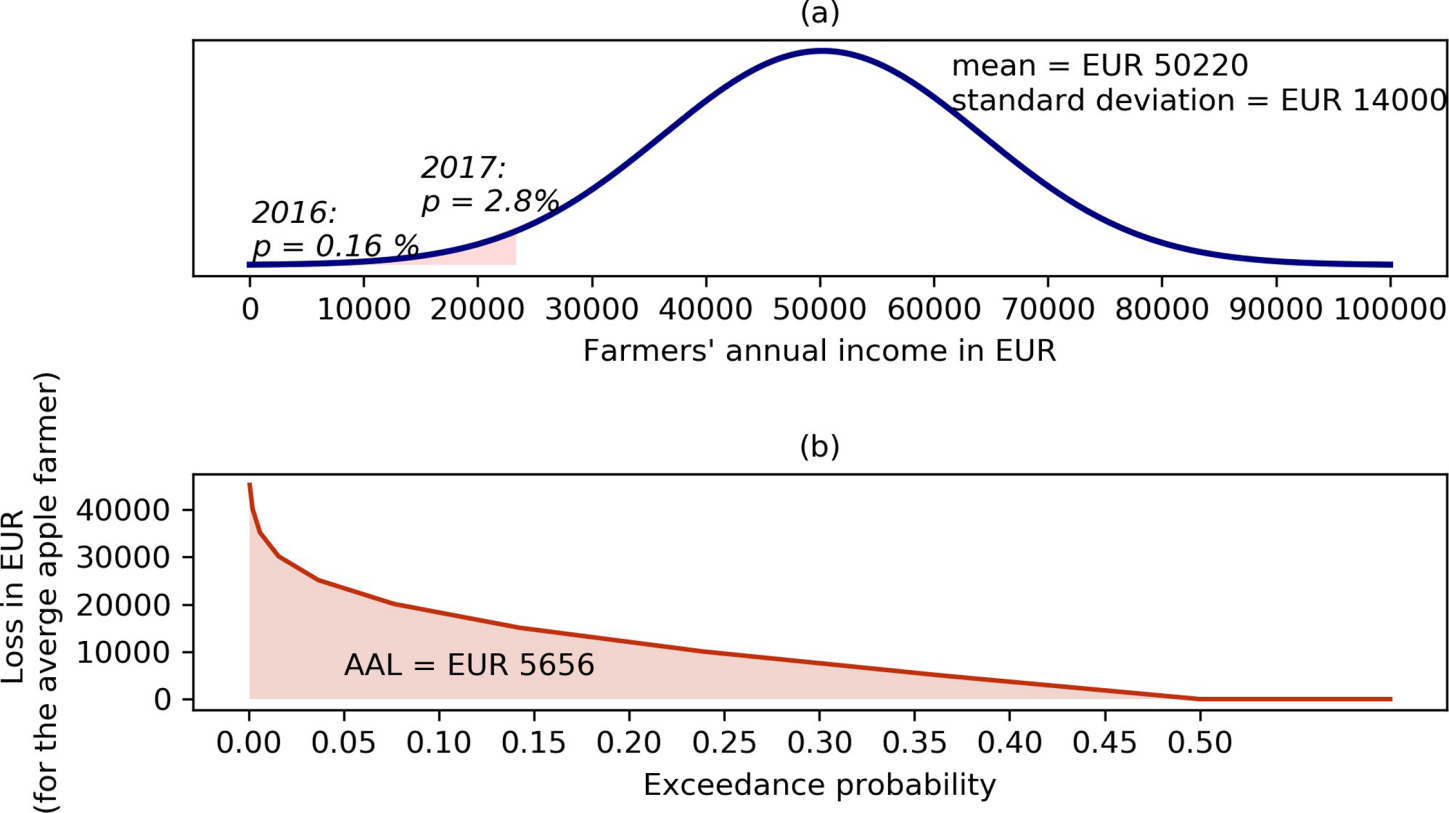
Panel (a) Distribution of apple farmers’ annual income. The dark red area refers to the probability of a reduction in yield as experienced in 2016. The light red shading shows the probability of an income reduction higher or equal to the one experienced in 2017. Panel (b): Exceedance probability curve describing the average annual income loss (AAL) faced by Styrian apple farmers. The numbers are inflation adjusted and correspond to EUR 2005 values.

The average annual income of Styrian apple farmers follows a normal distribution with a mean annual income of EUR 50,220 and a standard deviation of EUR 14,000 (see Fig 5A). These values are in line with the income situation in the Austrian agricultural sector [66]. Due to the small sample, a Shapiro-Wilk Test was applied to test for normal distribution, and confirmed. The Kolmogorow-Smirnow Test leads to the same conclusion.

The probability that farmers’ income is equal to or lower than the one earned in 2016 amounts to 0.16%. Hence, the spring frost as experienced in 2016, from an economic perspective, can be considered an extreme event with a return period lower than 1 in 500 years. Reducing the average income by 50% below the mean, the frost event in 2017 likewise had a severe impact. The probability of experiencing such a reduction in income amounts to around 3%; approximately a 30 year event.

Each annual income below the mean can be considered a loss. Fig 5B shows the exceedance probability curve indicating the probability that the annual loss exceeds a certain amount. The probability for example that the annual income loss exceeds EUR 15,000 is around 15%. The integral of the exceedance probability curve provides the average annual income loss (AAL) [54,55]. It amounts to EUR 5,656.

The total annual cost of each adaptation measure depends on the cultivated area it is supposed to protect, the number of frost events it is in operation, and the ratio between fixed and variable costs. The latter significantly differs across the described measures. While frost protection sprinkling as well as forced air circulation by wind machines have high fixed costs and low variable costs (ratio ≥ 3:1) artificial heating methods carry lower fixed costs but higher variable costs (ratio 1:1.6). Particularly those measures with a high share of variable costs (in particular anti-frost candles) may become excessively expensive when there is a high number of frost events and/or a large cultivated area to protect.

While we focus on all adaptation measures as listed in detail in S2 Table, the costs associated with the abandonment of unfavorable areas as well as a change of the composition of cultivated varieties are not included in the subsequent analysis. A refocus on hilly, less exposed locations would mean a reduction in apple production and additionally includes a substantial number of years without harvest in the initial growing phase of the new orchards. The same applies to cultivating new, more frost resistant varieties and is associated with a relatively high degree of uncertainty concerning the overall growing conditions and market acceptance. A quantification and monetization of such a long term process is beyond the scope of this study.

**Fig 6:**
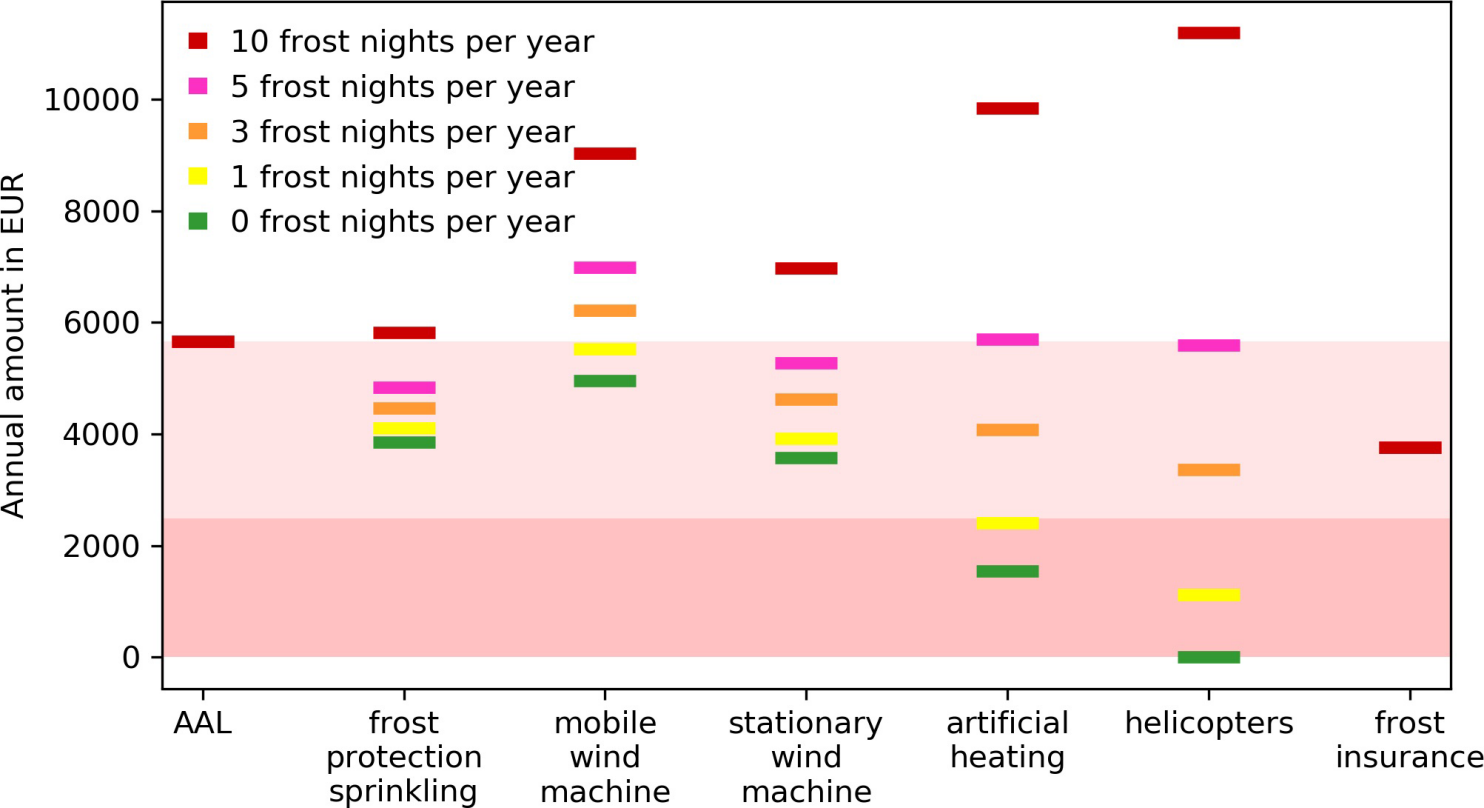
Average annual loss (AAL) versus annual costs of adaptation measures. The light red area shows the AAL when all possible sources of income losses as represented in Fig 5B are considered. The darker area refers to the AAL that are attributable to frost events only (at the frequency and damage ratio as experienced between 1978 and 2017; EUR 2,485).

Fig 6 contrasts the AAL (originating from past income variation) with the annual total cost of the different adaptation measures addressing primarily spring frost risk. The latter is shown for a varying annual frequency of frost events (0 to 10 nights). In a long-term evaluation, and assuming a stable frost risk, we can use the AAL as a yardstick to evaluate the economic appropriateness of adaptation measures. Adaptation measures to address single risks that cost more than the expected loss due to all risks will not pay off.

As indicated by the green bars (i.e., the case of 0 frost nights) frost protection sprinkling, forced air circulation- and artificial heating measures carry annual fixed costs ranging from EUR 2,500 (artificial heating) to EUR 5,000 (mobile wind machines). It is assumed that helicopters, if required, are provided either by the public sector or by contractors. They therefore do not carry any fixed costs.

Fig 6 reveals that the costs for frost insurance and frost protection sprinkling are least sensitive to the number of frost nights considered and steadily below or close to the AAL. Generally, one would expect the costs for insurance (at least if covering all risks) to be higher than the AAL, due to profit margins charged by the insurer [67]. Because premiums for bundled frost and hail insurance (which cover the main risks) currently are publicly subsidized, they are lower than the AAL. This makes them an interesting opportunity for apple producers to more actively manage their risks. One further advantage of insurance protection is that the insurance not only covers hail and frost damages but can also be extended to damages to hail nets caused by snow- and hailstorms. The advantages of frost protection sprinkling go beyond the mere provision of frost protection. The infrastructure may also be used for sprinkling during dry and/or hot spring and summer months. Additionally, it may also be used for applying fertilizers and repellents. Considering this diversity of application possibilities, frost protection sprinkling reduces multiple sources of potential income losses and therefore the yardstick of total AAL is a particular relevant one to use. From a pure cost perspective, stationary wind machines also seem like an appropriate way to reduce frost risk. Their application, however, is subject to limitations (e.g., noise, required building permit, limited to radiation frost, reduced efficiency with increasing distance to the crop [37]).

Measures with high variable costs (e.g., artificial heating or forced air circulations via helicopters) become less attractive the higher the frequency of frost events. Particularly, for more than five frost nights per year, the high share of variable costs for heating machines and helicopter flights leads to high total costs. Anti-frost candles are not included in Fig 6 due to their excessively high costs when applied to an area of 4 ha for several nights. The mere application to 1 ha carries costs of at least EUR 3,000 per frost night [68]. The corresponding numbers for Fig 6 are provided in S2 Table in the supporting information.

Considering the costs of the two most reasonable adaptation measures (frost protection sprinkling and insurance, see Fig 6), and assuming risk neutrality for the moment, these adaptation measures would pay for the average farmer if the likelihood of one frost night with an expected harvest loss of 50% is at or above 16% per year. Considering three consecutive frost nights and a harvest loss of 80% (as in 2016) an annual likelihood of 10% suffices.

Between 1978 and 2017, 5 frost related crop failures with income losses between 10% (1987) and 80% (2016) were experienced [69]. Assuming that the frost risk was stable within this 40 year period the annual probability of frost occurrence amounts to 12.5%. These spring frost events on average caused crop failures of around 40% [69]. Applying these numbers to the mean income suggests that the average annual loss caused directly by frost events in the last 40 years amounts to EUR 2,485. This is indicated by the dark red area in Fig 6. Hence, ignoring their additional benefit of income stabilization, at least for the average farmer currently neither frost protection sprinkling nor insurance is cost-effective. Yet, for the more exposed ones, obviously it is.

Assuming the connection between blocking and frost prevails also in a warmer climate, the advance of the blooming date shifts the vulnerable plant stage to a period in which there is a significant connection between blocking and frost days. For the current blooming period (day of the year 100–120), Fig 4 does not reveal any significant difference in the occurrence of frost days between randomly drawn days and days with blocking. When the blooming period shifts towards early April (day of the year 90–110), however, Fig 4 shows significantly more frost days under blocking conditions as compared to randomly drawn days. Moving from day of the year 100–120 to 90–110, the absolute number of frost days under blocking situations increases from 11 to 20, while the number of frost days under randomly drawn days increases from 9.2±3.9 days to 13.7±3.9 days. The difference of 13.7±3.9 random frost days to 20 blocking triggered frost days represents an increase of 46% with a range of 14% to 104%.

Evaluated at the probability of blocking (28% in day of the year 90–110) and assuming its stable occurrence, this suggests that the number of frost days increases by about 13% with a range of 4% to 29%, which we transfer to an equivalent increase in the likelihood of damage inducing frost episodes. Thus, conjoint considerations of blooming advancement and the influence of blocking conditions suggest that the annual probability of spring frost risk occurrence for south eastern Styrian farmers increases from 12.5% to 14% with a range of 13% to 16%. Note that blocking situations are likely to lead to a higher number of consecutive frost nights (e.g., April 25 to April 30, 2016), which further increases the damage potential. Consequently, adaptation measures will become economically beneficial for a larger fraction of apple producers in Styria. This holds even more, if farmers are not risk neutral, but stabilization of their income stream is valuable by itself.

## Discussion and conclusion

We combined a phenological sequential model with highly resolved climate projections for Austria, to evaluate the future frost risk for apple producers in south-eastern Styria. Our model projects a mean advance of apple blooming of –1.6 ± 0.9 days per decade (under RCP 4.5), shifting the bloom onset towards early April by the end of the 21st century.

As for the evolution of frost risk, mere consideration of our climate models’ estimates does not point towards any robust changes. The advancement of blooming towards early April, however, shifts the vulnerable plant stage to a period for which we find a strong connection between blocking and cold spells in our current climate. Assuming this connection prevails, this has the potential to increase frost risk to early blossom in the future.

Since climate models generally underestimate blocking and draw no robust conclusions about its future development, our ensemble of regional climate model projections likely shows a potential underrepresentation of frost risk. Observational studies, however, indicate an increased blocking occurrence and more extreme weather under recent climate change [62,63]. This suggests that the insights gained from the statistical observational analysis have vital implications for the assessment of future frost risk. The results we derived in detail for Styria are relevant for similar regions as well, including regions in Slovenia and northern Italy.

Besides blocking, also other weather situations can lead to late frost events, e.g., through radiative cooling. Overall, such impacts are highly variable and uncertain, and changes in their occurrence due to climate change are a topic of current research.

Beyond orchard trees, also natural trees face phenological changes due to climate change and past selection processes may not be suitable any longer to avoid spring frost damage [2,7,17]. The connection of blocking and cold spells in early spring should also be considered when studying climate change effects on natural vegetation.

For orchard cultures, as demonstrated by the impacts of the spring frost events in 2016 and 2017, the persistence and potential increase in frost risk requires adaptation measures to stabilize crop yields or ensure farmers’ income by other means. Physical and economic adaptation measures make the risks faced by apple producers explicit. Even though the annual costs for some measures and frost event frequencies are below the frost related average annual income risk faced by apple producers, they attach a price tag to the risk which is not apparent in the production costs when not implemented. Hence, once adaptation measures are implemented, production costs will increase. Overall, the sum of adaptation costs and residual damages, even if successfully minimized, represents additional climate related costs.

To share these emerging costs, coordinated action can be considered. For one, apple farmers could collectively install certain adaptation measures (e.g., frost protection sprinkling) and thereby spread investment costs. Production costs will nevertheless increase, yet at a smaller rate than via individual action. Regions may also politically decide to try and maintain certain levels of fruit production and to that end may transfer part or all of the increased risk to the public domain. Fractional–or up to full–absorption of additional costs could be achieved by public support. This could include, for example, the establishment of water reservoirs needed for widespread implementation of frost protection sprinkling. Also, publicly subsidized insurance schemes can help to increase the risk management capacity of farmers. While we have shown how and why frost risk could potentially increase for apple production, it is a political decision as to how the related increase of costs will be distributed between the individual (or sector) and the public.

## Supporting information

S1 Text Fulfillment of chilling requirements under the different climate models included. (DOCX)

S2 Text Thermal time model to determine apple blooming. (DOCX)

S1 Fig Value of the state of chilling on December 31 as described in S1 Text for each of the climate models included in the analysis and the two periods 1951 to 2000 (blue) and 2050 to 2099 (red). For none of the models a statistically significant change in the state of chilling between the two periods is found, as indicated by overlapping uncertainty ranges.
(TIF)

S2 Fig Thermal time model: Evolution of the blossoming day of apple in Weiz from 1951 to 2100 from the 5-year smoothed multi-model mean (green line) plus standard deviation (green shading) as well as from phenological observations from 1951 to 2006 (gray line). Potential frost damages (temperatures below –2.2°C occurring within 10 days after blossoming) are indicated for different regional climate models under RCP4.5 (colored dots). The dot size indicates how many grid points around Weiz are affected. Note that climate projections are not initialized with recent climate observations so that the variability of the simulated blossoming in the historical period does not match the observations.
(TIF)

S1 Table Regional climate models and global circulation models driving them. (DOCX)

S2 Table Investment and operational costs for each of the considered adaptation measures to cover a cultivated area of 4ha. (DOCX)
