# Supplementary material for: Spring frost risk for regional apple production under a warmer climate
Source: PLoS One. 2018 Jul 25;13(7):e0200201. doi: 10.1371/journal.pone.0200201 (PMC6059414; doi:10.1371/journal.pone.0200201)
Supplement: S1 Text — (DOCX) [file pone.0200201.s001.docx]

To test the applicability of a thermal time model (M1 in [1]), we compare the state of chilling (calculated according to Equations 1 and 2) on December 31 between a historical (1951 to 2000) and a future (2050 to 2099) period for all 11 models.

The chilling conditions show no statistically significant change (S1 Fig) as indicated by overlapping uncertainty ranges (even for the IPSL-CM5A-MR_RCA model), suggesting that the future temperature driven blooming advance will likely not be delayed due to insufficient chilling. This is in accordance with other studies that are comparable to our test region in Styria (more continental areas) [2–6]. In warmer regions (e.g., Mediterranean areas), however, a lack of chilling, will most likely lead to a delay of blooming [4–7]. Photoperiod, besides temperature the other main factor triggering and releasing dormancy, is supposed to exert its greatest effect only when chilling requirements are not completely fulfilled [8,9].

**References**

1. Chmielewski F-M, Blümel K, Henniges Y, Blanke M, Weber RWS, Zoth M. Phenological models for the beginning of apple blossom in Germany. Meteorologische Zeitschrift. 2011; 487–496. doi:10.1127/0941-2948/2011/0258

2. Vitasse Y, Schneider L, Rixen C, Christen D, Rebetez M. Increase in the risk of exposure of forest and fruit trees to spring frosts at higher elevations in Switzerland over the last four decades. Agricultural and Forest Meteorology. 2018;248: 60–69. doi:10.1016/j.agrformet.2017.09.005

3. Vitasse Y, François C, Delpierre N, Dufrêne E, Kremer A, Chuine I, et al. Assessing the effects of climate change on the phenology of European temperate trees. Agricultural and Forest Meteorology. 2011;151: 969–980. doi:10.1016/j.agrformet.2011.03.003

4. Campoy JA, Ruiz D, Egea J. Dormancy in temperate fruit trees in a global warming context: A review. Scientia Horticulturae. 2011;130: 357–372. doi:10.1016/j.scienta.2011.07.011

5. Luedeling E, Girvetz EH, Semenov MA, Brown PH. Climate Change Affects Winter Chill for Temperate Fruit and Nut Trees. PLOS ONE. 2011;6: e20155. doi:10.1371/journal.pone.0020155

6. Funes I, Aranda X, Biel C, Carbó J, Camps F, Molina AJ, et al. Future climate change impacts on apple flowering date in a Mediterranean subbasin. Agricultural Water Management. 2016;164: 19–27. doi:10.1016/j.agwat.2015.06.013

7. Legave JM, Blanke M, Christen D, Giovannini D, Mathieu V, Oger R. A comprehensive overview of the spatial and temporal variability of apple bud dormancy release and blooming phenology in Western Europe. Int J Biometeorol. 2013;57: 317–331. doi:10.1007/s00484-012-0551-9

8. Charrier G, Ngao J, Saudreau M, Améglio T. Effects of environmental factors and management practices on microclimate, winter physiology, and frost resistance in trees. Front Plant Sci. 2015;6: 259. doi:10.3389/fpls.2015.00259

9. Laube J, Sparks TH, Estrella N, Höfler J, Ankerst DP, Menzel A. Chilling outweighs photoperiod in preventing precocious spring development. Global Change Biology. 2014;20: 170–182. doi:10.1111/gcb.12360
