# Supplementary material for: Spring frost risk for regional apple production under a warmer climate
Source: PLoS One. 2018 Jul 25;13(7):e0200201. doi: 10.1371/journal.pone.0200201 (PMC6059414; doi:10.1371/journal.pone.0200201)
Supplement: S2 Text — (DOCX) [file pone.0200201.s002.docx]

The thermal time model (M1 in [1]), assumes that the chilling requirement is always fulfilled and converts daily mean temperatures above the base temperature for forcing $T_{BF}$ of 3.3°C to forcing rates according to Equation 3 and 4, always starting at $t_{1}$ with January 1. The optimized forcing requirement $F^{*}$ we find for the thermal time model amounts to 125 forcing units, which is comparable to the value reported for the region around Lake Constance by Chmielewski et al. (2011) [1].

As shown in S2 Fig, the thermal time model projects a similar trajectory of apple blooming onset as the sequential model. Obviously the standard deviation from the projections of the thermal time model is larger than the one from the sequential model, which is particularly related to the lower boundary. The reason is that in the thermal time model the beginning of apple tree blossom is not conditioned on the fulfillment of chilling requirements and can thus advance more strongly. This is particularly relevant for warmer climate projections.

**References**

1. Chmielewski F-M, Blümel K, Henniges Y, Blanke M, Weber RWS, Zoth M. Phenological models for the beginning of apple blossom in Germany. Meteorologische Zeitschrift. 2011; 487–496. doi:10.1127/0941-2948/2011/0258
