# Supplementary figures and images for: Spring frost risk for regional apple production under a warmer climate
Source: PLoS One. 2018 Jul 25;13(7):e0200201. doi: 10.1371/journal.pone.0200201 (PMC6059414; doi:10.1371/journal.pone.0200201)

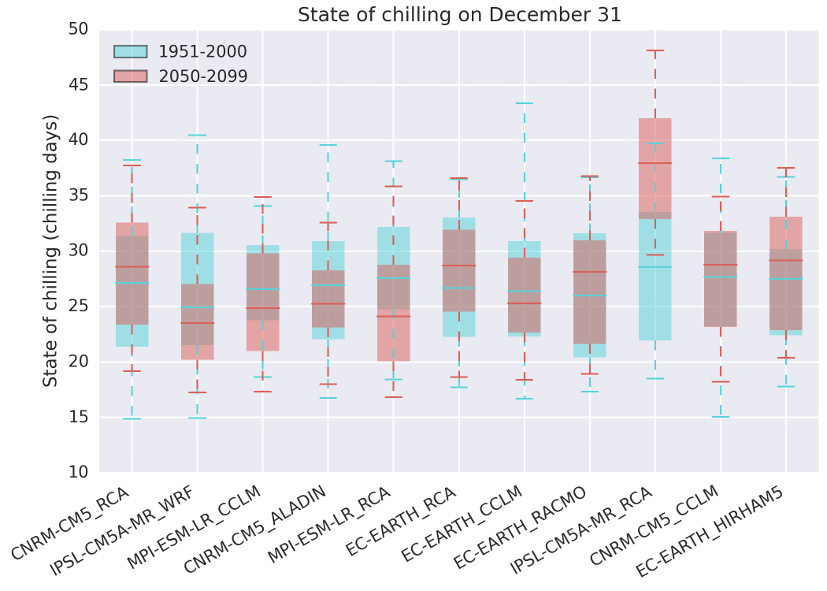

Supplement: S1 Fig — For none of the models a statistically significant change in the state of chilling between the two periods is found, as indicated by overlapping uncertainty ranges. (TIF) [file pone.0200201.s003.tif]

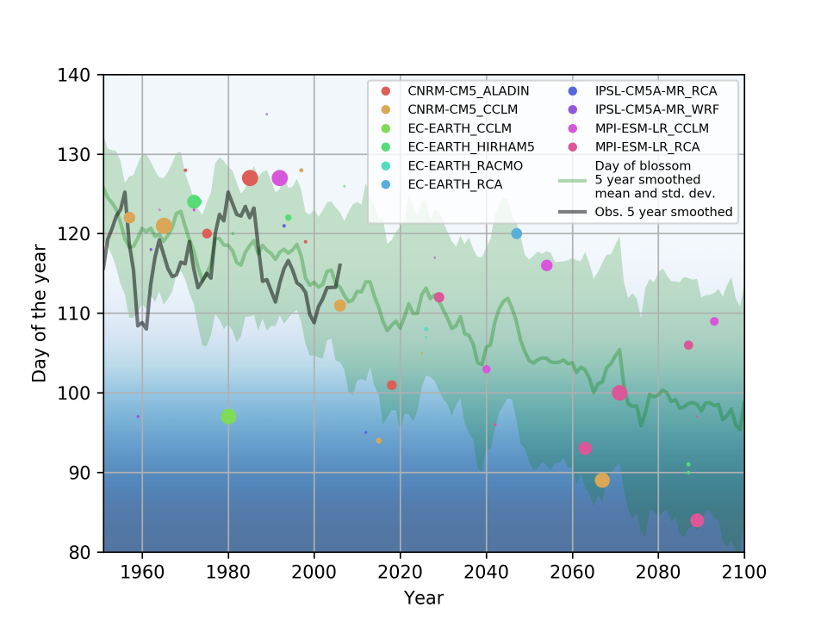

Supplement: S2 Fig — Potential frost damages (temperatures below –2.2°C occurring within 10 days after blossoming) are indicated for different regional climate models under RCP4.5 (colored dots). The dot size indicates how many grid points around Weiz are affected. Note that climate projections are not initialized with recent climate observations so that the variability of the simulated blossoming in the historical period does not match the observations. (TIF) [file pone.0200201.s004.tif]
