# Supplementary material for: Spring frost risk for regional apple production under a warmer climate
Source: PLoS One. 2018 Jul 25;13(7):e0200201. doi: 10.1371/journal.pone.0200201 (PMC6059414; doi:10.1371/journal.pone.0200201)
Supplement: S1 Table — (DOCX) [file pone.0200201.s005.docx]

S1 Table: Regional climate models and global circulation models driving them

| Global Circulation Models (GCMs) | | | Driven Regional Climate Models (RCMs) | |
| --- | --- | --- | --- | --- |
| Acronym | Reference publication | CMIP5 member | Acronym | Reference publication |
| CNRM-CM5^[[1]](#footnote-1)^ | [1] | r1i1p1 | RCA4  CCLM4-8-17  ALADIN | [2]  [3,4]  [5] |
| EC-EARTH | [6] | r12i1p1 r12i1p1 r1i1p1 r3i1p1 | RCA4  CCLM4-8-17  RACMO22E  HIRHAM5 | [2]  [3,4]  [7]  [8] |
| MPI-ESM | [9] | r1i1p1 | RCA4  CCLM4-8-17 | [2]  [3,4] |
| IPSL-CM5A | [10] | r1i1p1 | RCA4  WRF331F | [2]  [11] |

**References**

1. Voldoire A, Sanchez-Gomez E, Mélia DS y, Decharme B, Cassou C, Sénési S, et al. The CNRM-CM5.1 global climate model: description and basic evaluation. Clim Dyn. 2013;40: 2091–2121. doi:10.1007/s00382-011-1259-y

2. Patrick S, G JC, Ulrika W, Anders U, Stefan G, Ulf H, et al. The Rossby Centre Regional Climate model RCA3: model description and performance. Tellus A. 2011;63: 4–23. doi:10.1111/j.1600-0870.2010.00478.x

3. Böhm U, Kücken M, Ahrens W, Block A, Hauffe D, Keuler K, et al. CLM—the climate version of LM: Brief description and long-term applications. COSMO Newsletter. 2006;6: 225–235.

4. Rockel B, Will A, Hense A. The Regional Climate Model COSMO-CLM (CCLM). Meteorologische Zeitschrift. 2008; 347–348. doi:10.1127/0941-2948/2008/0309

5. Colin J, Déqué M, Radu R, Somot S. Sensitivity study of heavy precipitation in Limited Area Model climate simulations: influence of the size of the domain and the use of the spectral nudging technique. Tellus A. 2010;62: 591–604. doi:10.1111/j.1600-0870.2010.00467.x

6. Hazeleger W, Wang X, Severijns C, Ştefănescu S, Bintanja R, Sterl A, et al. EC-Earth V2.2: description and validation of a new seamless earth system prediction model. Clim Dyn. 2012;39: 2611–2629. doi:10.1007/s00382-011-1228-5

7. Meijgaard E van, Ulft LH van, Lenderink G, Roode SR de, Wipfler EL, Boers R, et al. Refinement and application of a regional atmospheric model for climate scenario calculations of Western Europe [Internet]. Wageningen: KVR; 2012 p. Report No.: KVR 054/12. Available: http://library.wur.nl/WebQuery/wurpubs/427097

8. Christensen OB, Christensen JH, Machenhauer B, Botzet M. Very High-Resolution Regional Climate Simulations over Scandinavia—Present Climate. J Climate. 1998;11: 3204–3229. doi:10.1175/1520-0442(1998)011<3204:VHRRCS>2.0.CO;2

9. Stevens Bjorn, Giorgetta Marco, Esch Monika, Mauritsen Thorsten, Crueger Traute, Rast Sebastian, et al. Atmospheric component of the MPI‐M Earth System Model: ECHAM6. Journal of Advances in Modeling Earth Systems. 2013;5: 146–172. doi:10.1002/jame.20015

10. Dufresne J-L, Foujols M-A, Denvil S, Caubel A, Marti O, Aumont O, et al. Climate change projections using the IPSL-CM5 Earth System Model: from CMIP3 to CMIP5. Clim Dyn. 2013;40: 2123–2165. doi:10.1007/s00382-012-1636-1

11. Skamarock C, Klemp B, Dudhia J, Gill O, Barker D, Duda G, et al. A Description of the Advanced Research WRF Version 3. 2008; doi:10.5065/D68S4MVH

1. Note, in the historical period the published CNRM-CM5 3D data are mixed with inconsistent sea surface temperature fields (Samuel Somot, CNRM; Jan 30, 2018; personnel communication). Since this has only a minor climate change effect on temperature in the Alpine region, we decided with all caution to keep the CNRM-CM5-based EURO-CORDEX projections in our analysis. [↑](#footnote-ref-1)
