# Supplementary material for: Spring frost risk for regional apple production under a warmer climate
Source: PLoS One. 2018 Jul 25;13(7):e0200201. doi: 10.1371/journal.pone.0200201 (PMC6059414; doi:10.1371/journal.pone.0200201)
Supplement: S2 Table — (DOCX) [file pone.0200201.s006.docx]

| **Adaptation measure** | **Assumed investment costs per year (in EUR)**^[[1]](#footnote-1)^ | **Assumed operational costs (in EUR)**^[[2]](#footnote-2)^ | **Source** |
| --- | --- | --- | --- |
| **Frost protection sprinkling** | EUR 3861 | Maintenance costs: EUR 200/100h  Hourly wages: EUR 15/h  Energy costs: EUR 15/h | [1,3,4] |
| **Mobile wind machine** | EUR 4951 | Fuel consumption: 30 L/h and EUR 0.8/L  Maintenance costs: EUR 200/100h  Transport costs: EUR 30/h  Hourly wages: EUR 15/h | [1,4] |
| **Stationary wind machine** | EUR 3577 | Fuel consumption: 40 L/h and EUR 0.8/L  Maintenance costs: EUR 200/100h | [1,4] |
| **Artificial heating** | EUR 1547 | Fuel Consumption: EUR 45L/h and EUR 0.8/L  Maintenance costs: EUR 200/100h  Hourly wages: EUR 15/h  Transport costs: EUR 30/h | [4,5] |
| **Anti-frost candles** | - | Candles: EUR 12000  Hourly wages: EUR 15/h | [6] |
| **Helicopter** | - | EUR 1120 per frost night | [5] |
| **Insurance** | - | EUR 3758 per year^[[3]](#footnote-3)^ | Personal communication with Josef Kurz from the Austrian Hail Insurance Company |

**References**

1. Baumann F. Vermeidung von Spätfrösten bei Kernobst durch den Einsatz von Windmaschinen am Beispiel des Prototyps “Freilandwind BSR 5.” University of Applied Sciences Osnabrueck 2014. 2014; Available: http://freilandwind.de/wp-content/uploads/2014/10/Bachelorarbeit1.pdf

2. Bundesministerium für Bildung. Anlagenkennzahlen mit den Nutzungsdauerwerten. 2004; Available: https://www.bmb.gv.at/ministerium/rs/2010_27_beilage2_20004.pdf?5te7hi

3. Snyder RL, Melo-Abreu JP. Frost protection: fundamentals, practice and economics. Volume 1 [Internet]. FAO; 2005. Available: https://www.repository.utl.pt/handle/10400.5/4727?locale=en

4. Spuhler M, Neuwald DA. Frost 2016. Kompetenzzentrum Obstbau Bodensee Arbeitsbereich Ernte Lager Fruchtqualität. 2016;Universität Hohenheim, Fachgebiet: Ertragsphysiologie der Sonderkulturen.

5. Schwappach P. Frostabwehr im Weinbau. Bayrische Landesanstalt für Weinbau und Gartenbau. 2012; Available: http://docplayer.org/44893509-Frostabwehr-im-weinbau.html

6. Luttenberger W, Mazelle M. Aktuelle Hinweise zur Frostabwehr im Obst- und Weinbau. Landwirtschaftskammer Steiermark. 2017; Available: https://stmk.lko.at/aktuelle-hinweise-zur-frostabwehr-im-obst-und-weinbau+2500+2551422

1. Due to infrequent operation, for each of the measures an average operating life time of 20 years is assumed [1,2]. [↑](#footnote-ref-1)
2. The actual variable cost depends on the number of frost nights experienced in a given year. [↑](#footnote-ref-2)
3. Exemplary average premium for one municipality in south-eastern Styria (St. Ruprecht). The actual premium faced by individual farmers depends on the amount insured, past damage record, chosen insurance option and location, with a reported range between EUR 2488 and EUR 5028 (for 4 ha in this particular municipality). [↑](#footnote-ref-3)
